# Improvement of Negative Psychological Stress Response in Elderly Patients With Femoral Neck Fracture by Integrated High-Quality Nursing Model of Medical Care

**DOI:** 10.3389/fsurg.2022.859269

**Published:** 2022-03-24

**Authors:** Qun Li, Yin Wang, Xiang Shen

**Affiliations:** Orthopaedics Department, The Fourth Hospital of Changsha (Affiliated Changsha Hospital of Hunan Normal University), Changsha, China

**Keywords:** femoral neck fracture, integration of medical care, quality care, medical care, elderly patients

## Abstract

**Objective:**

The objective of this study was to explore the nursing effect and negative psychological stress response of elderly patients with femoral neck fracture by applying the high-quality nursing mode of medical care.

**Methods:**

A total of 130 elderly patients with femoral neck fractures hospitalized in our hospital from January 2020 to June 2021 were randomly divided into the control group and observation group, with 65 patients in each group. The control group adopted the conventional nursing mode, while the observation group adopted the high-quality nursing mode of medical care. The observation indexes selected in this study are nursing satisfaction, hip flexion activity on the 1, 15, and 30 days after the operation, the time when the affected limb was lifted off the bed actively, and the anxiety and depression of patients.

**Results:**

On the 1, 15, and 30 days after the operation, there were statistically significant differences between the two groups in hip flexion activity and the time when the affected limb was lifted off the bed (*P* < 0.05). The nursing satisfaction of the observation group was 95.38%, which was statistically significant compared with the 80.00% of the control group (*P* < 0.05). After treatment, the self rating depression scale (SDS) and self rating anxiety scale (SAS) scores in the observation group were lower than those in the control group (*P* < 0.05).

**Conclusion:**

The high-quality nursing model of medical care can effectively promote the rehabilitation of elderly patients with femoral neck fracture, reduce the negative psychological stress reaction of patients, and improve nursing satisfaction, which has important application value and guiding significance for the nursing of patients with femoral neck fracture.

## Introduction

Femoral neck fracture is a common and frequently-occurring disease in clinics, with the highest incidence rate among middle-aged and elderly patients, accounting for 3.58% of all fractures. Femoral neck fracture is a serious type of fracture and it takes a long time to reconstruct or recover the fracture site after an operation, which has brought serious adverse effects to the patients' normal work and life (1, 2). Elderly patients with femoral neck fractures will feel obvious pain at the broken end of the fracture, especially when moving the injured limbs. Pain has a variety of adverse effects on the body, which not only form complex physiological reactions and affect the rehabilitation exercise of patients but also lead to negative emotions of patients (3).

At present, the treatment of femoral neck fracture mainly uses artificial hip joint replacement, which can relieve the pain of patients as soon as possible, reduce the damage to patients' bodies, and restore normal life. However, the curative effect of the operation is closely related to good nursing care, so it is necessary to strengthen nursing management during the perioperative period to promote the early recovery of patients. Medical integration refers to the process in which doctors and nurses provide medical care services to patients with certain professional knowledge and ability through open coordination and communication on the premise of equality, independence, mutual respect, and trust (4, 5). The integrated high-quality nursing model of medical care is a new type of whole-course management model, which is widely used in clinical nursing. However, there are few in-depth reports on the application of the integrated high-quality nursing model of medical care in the perioperative period of elderly patients with femoral neck fractures. At present, the nursing work for elderly patients is still based on the traditional medical model of “independent evaluation of medical care, doctors' orders, and nurses' orders,” which lacks the cooperation and communication between doctors and nurses. This study focuses on the in-depth analysis of the high-quality nursing model of medical care for elderly patients with femoral neck fracture, which is reported as follows.

## Data And Methods

### General Information

A total of 130 elderly patients with femoral neck fractures hospitalized in our hospital from January 2020 to June 2021 were selected and divided into the control group and the observation group with 65 patients in each group according to the random number table method. Inclusion criteria: age ≥60 years old; imaging diagnosis is femoral neck fracture; postoperative hospital stay > 5 days; awareness, communication skills, and no surgical contraindications. Exclusion criteria: accompanied by serious heart, lung, liver, kidney, and other diseases; Complicated with neurological, immune, and endocrine system diseases; Severe preoperative malnutrition; Poor foundation, unable to tolerate surgery. This study was approved by the Ethics Committee of our hospital and the patients' and their families informed consent.

### Research Methods

#### Nursing Method of the Control Group: The Patients Were Nursed by Conventional Nursing Mode

Doctors and nurses made ward rounds every day to make treatment and care plans, respectively, and implement the plans, respectively, such as fasting and water deprivation for 6 h after operation, general diet for 6 h, absolutely lying in bed after the operation, with the affected limb higher or slightly higher than the heart level, maintaining knee joint flexion of 20–30 degrees, performing metatarsophalangeal joint and toe joint movement of the affected limb after anesthesia recovery after the operation, and performing doctor-permitted movement after except fixation 6–8 weeks after the operation.

#### Nursing Method of Observation Group: Adopt the High-Quality Nursing Mode of Medical Care

1. Establish a medical and nursing integrated diagnosis and treatment team: its members include doctors and nurses at all levels, and the department heads and head nurses are responsible for it. In the observation group, the medical staff in the group jointly completed the medical history collection, physical examination, and condition evaluation. The doctors wrote inpatient medical records and nurses set up nursing logs.
2. Integrated medical ward rounds: doctors and nurses make ward rounds every morning and evening to learn about the patient's condition and the implementation of treatment. The degree of limb swelling, self-care ability of patients in daily life, implementation of doctor's advice, adverse drug reactions reported by doctors, etc. design the record of joint medical rounds, convey the opinions of doctors to all nurses in this group, and adjust the functional exercise and nursing plan.
3. Painless handling. Doctors and nurses cooperate with each other in transportation. The doctors are responsible for pulling the affected limb and choosing different numbers of nurses according to the patient's weight. Nurses respectively support the patient's torso and the contralateral lower limbs so that the patient can maintain the abduction neutral position. The doctor gave the orders, and at the same time, the doctor lifted the patient. Under the doctor's traction, the affected limb is always on the same horizontal line as the patient's longitudinal axis. After the operation, the doctors and nurses put the patient together in a comfortable position.
4. Quality environmental care. For patients hospitalized for a long time, the ward environment should be kept clean to prevent hospital infection. Ventilation keeps the air in the ward fresh every day. A total of 84 kinds of disinfectants are used to wipe the table and floor every day.
5. Integration of medical care and functional exercise: Individualized functional exercise programs for patients should be developed based on the general situation of patients, osteoporosis, and self-care ability in daily life. Under the guidance of the doctor and the assistance of nurses, the patients' families were instructed to do the decompression exercise to reduce the pressure of the calf and thigh muscle from the patient's heel 6 h after the operation, three times a day, each time for 15 min, and 48 h after the operation, the patients were treated with double lower limb pneumatic pump. Exercise should be carried out step by step under the guidance of a doctor, and should not be too hasty to avoid injury again.
6. Psychological care. According to the patient's education level and knowledge receiving ability, choose a suitable way to explain the disease and the correlation between postoperative rehabilitation-related knowledge, emotion, and postoperative recovery, so that patients can do a good job in ideological work Tell the patients to keep a good attitude and insist on rehabilitation training, which will help to improve the rehabilitation effect. Doctors and nurses must actively answer the problems existing in the patients' treatment and rehabilitation. Encourage the patients to maintain communication with the outside world and win the support of family members, especially children or spouses of patients. Help the patients to establish reasonable cognition, reduce negative emotions, and build their confidence to cooperate with the treatment and rehabilitation exercises. During the implementation of psychological nursing, the nurses implemented the corresponding functional exercise measures for the patients and signed them to ensure that the nursing measures were in place.

### Observation Indicators

The observation indexes selected in this study are nursing satisfaction, hip flexion activity on the 1, 15, and 30 days after the operation, the time when the affected limb is lifted off the bed actively, and the anxiety and depression of patients. Among them, nursing satisfaction was measured by the self-made questionnaire of clinical nursing satisfaction of patients in our hospital. Out of 100, >85 is very satisfactory, 70–85 is satisfactory, 60–70 is fair, <60 is unsatisfactory, and satisfaction = very satisfactory rate+satisfactory rate. The content validity index of the self-made questionnaire in our hospital was 0.83, and the α coefficient of Kehlenbach was 0.816, with good reliability and validity. The self-rating anxiety scale (SAS) and self-rating depression scale (SDS) were used to evaluate the anxiety and depression of patients before nursing (when entering the group) and after nursing (30 days after the operation). The cut-off value of the SAS scale is 50 points, 50–59 points for mild anxiety, 60–69 points for moderate anxiety, and 69 points or more for severe anxiety. The demarcation value of the SDS scale is 53 points, 53–62 points for mild depression, 63–72 points for moderate depression, and 72 points or more for severe depression.

### Statistical Methods

The SPSS22.0 software (IBM Corp., Armonk, NY, USA) was used to process the experimental data. The experimental data were in accordance with the normal distribution. The measurement data were expressed by the mean SD ($\bar{x}$ **±** s), and the counting data was expressed by (%). The *t*-test analysis was used for pairwise comparison of measurement data between groups, and the χ^2^ test was used for counting data. The test level is α = 0.05, and the difference is statistically significant when *P* < 0.05.

## Results

### Comparison of General Data of Two Groups of Patients

There was no significant difference in general data such as gender and age between the two groups (*P* > 0.05). As shown in Table 1.

**Table 1:** Comparison of general information of two groups of patients.

**Group**	**Number**	**Gender**		**Age (years)**	**Cause of injury**			**Fracture site**		
		**Male**	**Woman**		**Traffic accident**	**Wrestling**	**Fall down**	**Other**	**Left side**	**Right side**
Control group	65	34	31	71.02 ± 3.27	15	34	6	10	39	26
Observation group	65	35	30	70.94 ± 3.11	19	32	8	6	37	28
*t/χ^2^* value	0.031	0.143	1.817	0.127
*P* value	0.860	0.887	0.611	0.722

### Comparison of Hip Joint Flexion Range of Motion Between Two Groups

There was a statistically significant difference in the hip joint flexion range of motion of the patients in the observation group is higher than that of the patients in the control group on the 1, 15, and 30 days after the operation (*P* < 0.05). As shown in Table 2.

**Table 2:** Comparison of flexion range of hip joint between two groups.

**Group**	**Number**	**Hip joint flexion range of motion (** **°** **)**		
		**1d**	**15d**	**30d**
Control group	65	46.59 ± 7.39	61.83 ± 6.03	81.21 ± 5.29
Observation group	65	55.80 ± 6.27	74.25 ± 7.24	105.28 ± 9.13
*t* value		7.662	10.627	18.391
*P* value		<0.001	<0.001	<0.001

### Comparison of Active Lifting Time of Affected Limb From Bed Between Two Groups of Patients

**Figure 1:**
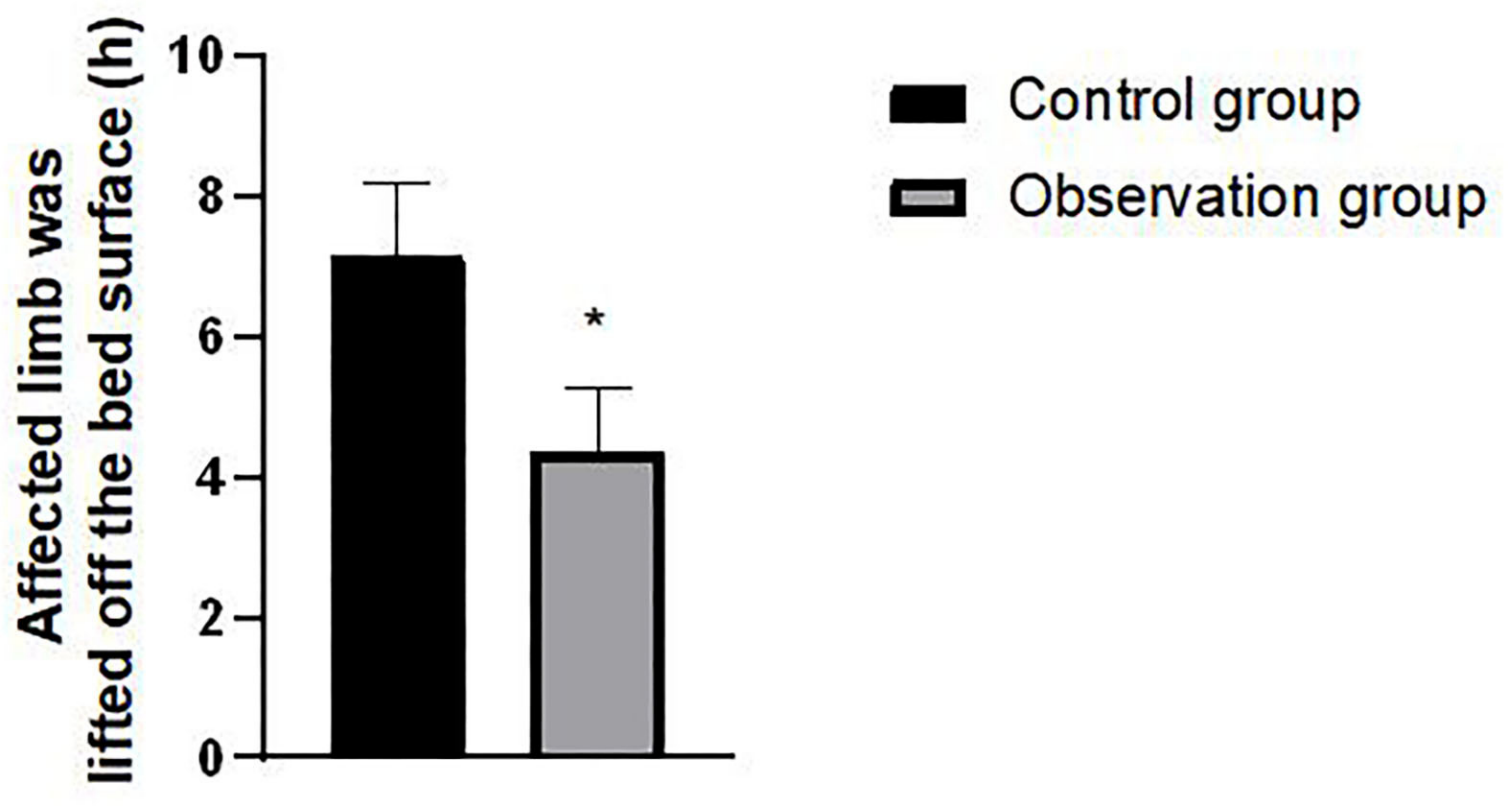
Comparison of active lifting time of affected limb from bed between two groups of patients. Compared with the control group, *t = 16.465, *P* < 0.001.

There was a statistically significant difference in the time when the affected limb was lifted off the bed surface between the two groups (*P* < 0.05). As shown in Figure 1.

### Comparison of Nursing Satisfaction Between Two Groups of Patients

**Figure 2:**
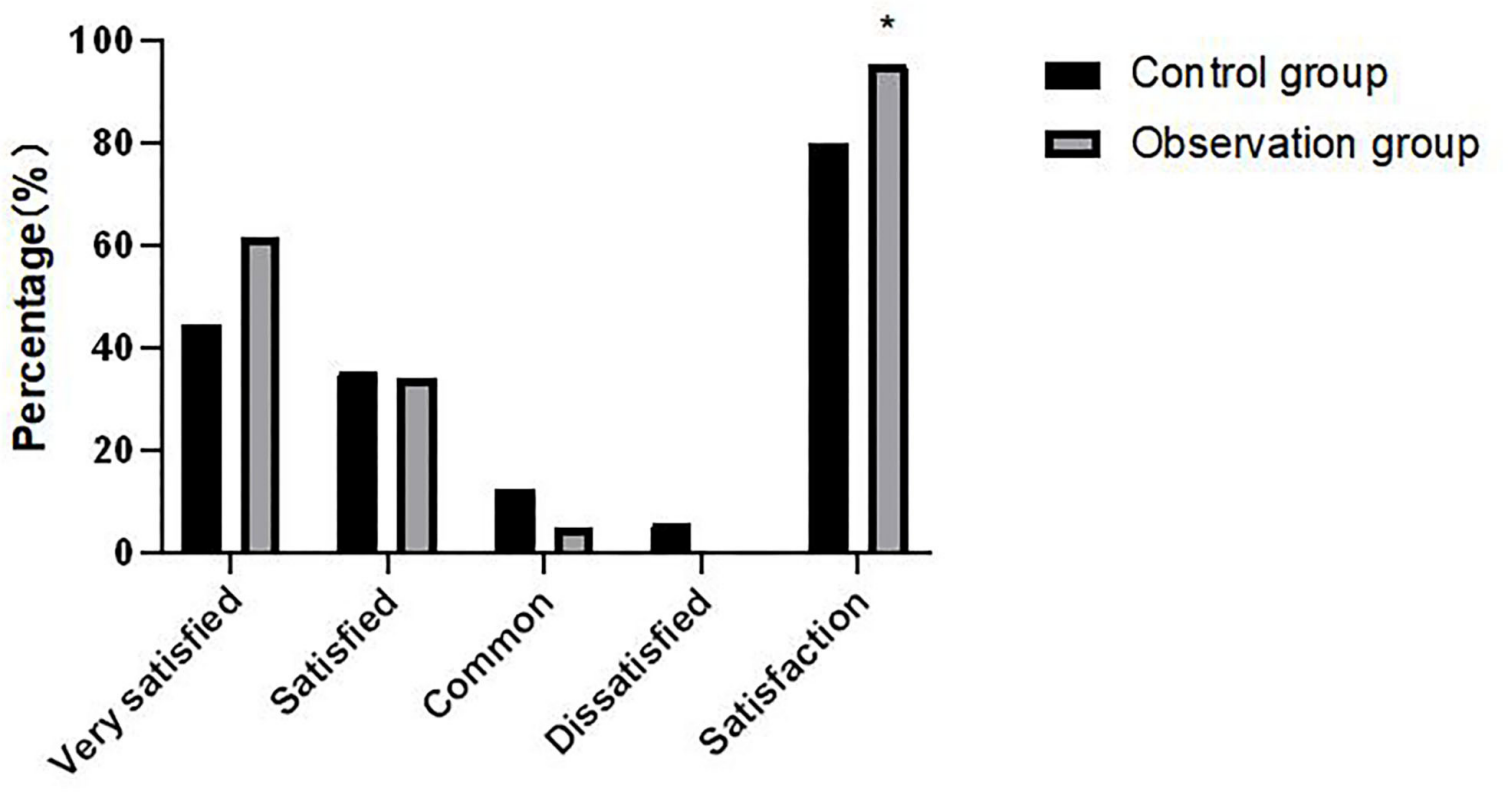
Comparison of nursing satisfaction between two groups of patients. Compared with the control group, *χ^2^= 9.049, *P* = 0.029.

The nursing satisfaction of the observation group was 95.38%, which was statistically significant compared with the 80.00% of the control group (*P* < 0.05). As shown in Figure 2.

### Comparison of the SDS and SAS Scores Between the Two Groups Before and After Treatment

After treatment, the SDS and SAS scores of the patients in the observation group were lower than those in the control group, and the difference was statistically significant (*P* < 0.05). As shown in Table 3.

**Table 3:** Comparison of the SDS and SAS scores between the two groups before and after treatment.

**Group**	**Number**	**SDS score**		**SAS score**	
		**Before treatment**	**After treatment**	**Before treatment**	**After treatment**
Control group	65	60.15 ± 7.03	49.37 ± 6.19	57.92 ± 6.31	47.29 ± 5.02
Observation group	65	59.23 ± 6.71	32.85 ± 5.93	59.12 ± 6.69	31.94 ± 6.18
*t* value		0.763	15.537	1.052	15.543
*P* value		0.447	<0.001	0.295	<0.001

## Discussion

With the arrival of China's aging society, the incidence of osteoporosis is getting higher and higher, and the incidence of femoral neck fracture is on the rise, which has aroused great concern in medical circles. For the treatment of this disease, surgery is widely favored by patients and doctors for its definite curative effect and low incidence of complications (6). A large number of clinical studies have proved that good nursing intervention to patients with femoral neck fracture can promote postoperative rehabilitation of elderly patients (7, 8).

The high-quality nursing mode of medical care integrates the etiology, manifestation, prevention, rehabilitation, and nursing of elderly patients with femoral neck fracture, finds out the changes of patients' condition in time, and breaks the original pattern of the doctor-patient nurse-patient parallel (9). The high-quality nursing mode of medical and nursing integration has set up a brand-new three-dimensional integrated working pattern of doctors, nurses, and patients, implements the joint ward rounds of doctors and nurses, and made targeted planning of perioperative nursing measures of patients, making the cooperation between doctors and nurses more systematic (10).

The high-quality nursing mode of medical integration can help nurses understand the key points of the patients' functional exercise. Doctors and nurses can jointly guide patients to carry out individualized functional exercises to ensure the safety and effectiveness of functional exercise. At the same time, targeted education should be taken to improve the quality of functional exercise and patient compliance according to the patient's mastery (11, 12). The observation group implemented the high-quality nursing mode of integrated nursing of medical staff and worked out the early functional exercise plan of the affected limb to help patients recover. The observation indexes were all better than those of the control group. Especially on the 1, 15, and 30 days after the operation, the improvement effect of hip flexion activity, the time for the affected limb to lift off the bed surface, and other indexes are better than that of the control group, which provides powerful help for achieving the ideal rehabilitation effect. It can be seen that the high-quality nursing model of integration of medical care and nursing adopted in this study has achieved an ideal nursing effect, shortened the recovery time of patients, and laid a solid foundation for them to resume a normal life.

In the process of implementing the high-quality nursing model of medical integration, patients are also involved, and the relationship between doctors and patients is transformed into a new cooperative relationship (13). This study shows that the nursing satisfaction of the observation group is significantly higher than that of the control group. In the process of patient treatment, the medical staff really take the patients as their own responsibilities and obligations and do their duty to the patients in charge, so that patients can enjoy high-quality and satisfactory care, meet the knowledge needs of patients and their families, further deepen and highlight the connotation of high-quality medical services, and improve patient satisfaction (14). It has important application value and guiding significance for the treatment of patients with femoral neck fractures or fractures in other parts of lower limbs.

Although the operation has a good effect on femoral neck fracture, because most of the patients are elderly, they have poor psychological endurance, slow reaction, unclear language expression, lack of understanding of diseases and operations knowledge, and worry too much that the fracture site will return to normal after the operation, which will easily lead to negative psychology such as anxiety and depression, etc. If they are not dealt with in time and effectively, it will affect the postoperative recovery of patients (15, 16). This study showed that after treatment, the SDS and SAS scores of the patients in the observation group were significantly lower than those in the control group after treatment. It shows that the high-quality nursing mode of integrating medical care with nursing care has a certain effect on improving patients' negative emotions. From the patient's point of view, the integrated high-quality nursing model, clear division of labor between doctors and nurses, and personalized high-quality nursing service greatly improve the nursing quality. Compared with simple clinical treatment and routine nursing, it can relieve patients' negative psychological stress and promote their rehabilitation (17, 18).

To sum up, the high-quality nursing model of medical and nursing integration can effectively promote the rehabilitation of elderly patients with femoral neck fracture, reduce the negative psychological stress reaction of patients and improve nursing satisfaction, which has important application value and guiding significance for the nursing of patients with femoral neck fracture.

## Data Availability Statement

The original contributions presented in the study are included in the article/supplementary material, further inquiries can be directed to the corresponding author.

## Ethics Statement

The studies involving human participants were reviewed and approved by the Medical Ethics Committee of the Fourth Hospital of Changsha. The patients/participants provided their written informed consent to participate in this study.

## Author Contributions

QL, YW, and XS made equal contributions to this study, including study design, inclusion of cases, evaluation of results, data statistics, and article writing. XS was the supervisor of the entire study. All authors contributed to the article and approved the submitted version.

## Conflict of Interest

The authors declare that the research was conducted in the absence of any commercial or financial relationships that could be construed as a potential conflict of interest.

## Publisher's Note

All claims expressed in this article are solely those of the authors and do not necessarily represent those of their affiliated organizations, or those of the publisher, the editors and the reviewers. Any product that may be evaluated in this article, or claim that may be made by its manufacturer, is not guaranteed or endorsed by the publisher.
